# Case-Control Study of Postoperative Blood Pressure in Patients with Hemorrhagic Complications after Laparoscopic Sleeve Gastrectomy and Matched Controls

**DOI:** 10.1007/s11695-017-2569-x

**Published:** 2017-01-30

**Authors:** Michał Robert Janik, Tomasz Rogula, Piotr Kowalewski, Maciej Walędziak, Maciej Matłok, Jakub Brągoszewski, Andrzej Kwiatkowski, Krzysztof Paśnik

**Affiliations:** 1 Bariatric and Metabolic Surgery - Geauga Medical Center, Case Western Reserve University School of Medicine, 10900 Euclid Ave. T402, Cleveland, OH 44106 USA; 2 Department of General, Oncologic, Metabolic and Thoracic Surgery, Military Institute of Medicine, Szaserów, 04-141 Warszawa, Poland; 3 Faculty of Medicine and Health Sciences, Jan Kochanowski University, Kielce, Poland; 4 2nd Department of Surgery UJCM, Jagiellonian University, ul. Kopernika 21, 31-501 Kraków, Poland

**Keywords:** Bariatric surgery, Sleeve gastrectomy, Bleeding, Complications, Blood pressure, Postoperative hypertension, Hemorrhagic complications

## Abstract

**Introduction:**

Laparoscopic sleeve gastrectomy is associated with a moderate risk of hemorrhagic complications (HC). There is a debate regarding the relationship between HC and high blood pressure in postoperative period.

**Aim:**

The aim is to clarify whether the postoperative blood pressure is an independent risk factor for hemorrhagic complications after laparoscopic sleeve gastrectomy.

**Methods:**

Medical records of 522 patients were reviewed. A case-control study of postoperative blood pressure was undertaken in patients with bleeding after LSG and matched controls. Patients who required surgical revision, due to the hemorrhagic complications within 72 hours, were identified as the cases. Controls were matched (1:1) with cases by age (±1 year), gender (female versus male), staple line reinforcement (running suture versus haemostatic clips) and surgeon’s experience (>50 or <50 LSG procedures per year). 12-hour postoperative blood pressure was recorded.

**Results:**

17 patients after LSG with HC in postoperative period were matched with 17 controls. Patients who experienced hemorrhagic complications after LSG had non statistically significant decreased mean systolic blood pressure (mmHg) in 12 hours observation (130.7 ± 12.9 versus 139.1 ± 10.8); *p* = 0.15; mean difference − 11.6 (95% CI -29.5 – 6.1). Mean 12 hour diastolic pressure was also comparable. The detailed analysis of controls revealed a significantly higher systolic blood pressure measurements in 5th and 11th hour postoperatively, as well as higher diastolic blood pressure in 12th hour postoperatively. However, the differences were not clinically significant.

**Conclusion:**

Compared with closely matched control subjects, patients with HC after LSG have decreased systolic blood pressure without clinical significance.

## Introduction

Laparoscopic sleeve gastrectomy (LSG) is one of the most popular bariatric procedures [1]. The procedure was introduced as a first step of biliopancreatic diversion with duodenal switch [2]. A decade later sleeve gastrectomy became a stand-alone procedure [3]. The LSG gained popularity amongst surgeons due to its simplicity. The procedure is well accepted by patients. The results of LSG are generally good [4, 5]. LSG and Roux-Y Gastric Bypass are equally effective in terms of excess weight loss (%EWL) [6]. However, LSG is associated with the risk of following serious adverse events: gastric leakage, hemorrhagic complications and sleeve stenosis. Despite the different methods of staple line reinforcement [SLR] the incidences of bleeding and leakage occur in 1–5% [7]. Even 3% of patients after LSG require reoperation [8]. The majority of papers was focused on the problem of gastric leaks, whereas the issue regarding hemorrhagic complications is not explored enough. Jossart et al. stated that hemorrhage from the staple line or short gastric vessels is more likely to occur with episodes of hypertension [9]. However, there is no of evidence that high blood pressure in postoperative period may contribute to increased risk of bleeding. This case-control study of postoperative blood pressure was undertaken in patients with bleeding after LSG and matched controls.

The aim was to clarify whether the postoperative blood pressure is an independent risk factor for hemorrhagic complications after laparoscopic sleeve gastrectomy.

## Methods

### Study Design

We performed a case-control study. The medical records of 522 patients who underwent laparoscopic sleeve gastrectomy as a primary bariatric procedure were analyzed. All patients underwent surgery from January 2013 and May 2015 in a single high volume bariatric centre (> 300 laparoscopic bariatric procedures per year). The same cohort was used to develop prediction model for HC and construct risk calculator called SLEEVE BLEED [10]. Antiplatelet therapy and NSAIDs were discontinued a week before the surgery. The deep vein thrombosis prophylaxis was the same for every patient. Since every case had estimated Caprini score of 4 points or more we combined mechanical methods (graduated compression stockings) with pharmacology (low-molecular weight heparin administered 12 h preoperatively and postoperatively).

### Participants - Subjects

Patients who required surgical revision due to the hemorrhagic complications within 72 h were identified as the cases. Hemorrhagic complications included bleeding from different sources, including staple line, omentum, short gastric vessels and abdominal wall as well as the presence of large hematoma [9]. The postoperative bleeding became apparent within 4–24 h depending on its severity. We did not observe a full hypovolemic shock in any of the cases. The diagnosis was based on drainage output, drop in the red blood count and sonogram (excessive amount of fluid in the peritoneum). There was no fixed threshold for reoperation. Every decision was made individually, based on previously mentioned clinical features.

We found 21 cases of patients with hemorrhagic complications. Mean age (years) of patients was 43.9 ± 10.4 ranging from 23 to 57. 38% were female and 62% were male. The incidence of bleeding in our material was 4.03%. The same database was used in development of risk model for hemorrhagic complications.

### Participants - Normal Controls

Controls subjects were taken retrospectively from the rest of patients who underwent LSG from January 2013 to May 2015 and did not experienced any of following adverse events: hemorrhagic complications, gastric leakage or sleeve stenosis. All controls identified as potential matched controls for the cases.

### Matching

Controls were matched with cases by age (±1 year), gender (female versus male), staple line reinforcement (running suture versus haemostatic clips) and surgeon’s experience (>50 or <50 LSG procedures per year). A control subject was selected for each patient with HC using algorithm described by Kawabata (1:1 matching procedure) [11]. Cases for whom we could not identify suitable matching control subjects were excluded from the study.

### Surgical Technique and Post-Operative Management

In Laparoscopic Sleeve Gastrectomy five trocars were used. The dissection of the greater curvature was started 3 to 4 cm from the pylorus. The gastrocolic ligament was cut to the angle of His. The left diaphragmatic crus was exposed. A 36-French bougie was used to calibrate the sleeve. A stapler was introduced and fired consecutively along the length of the bougie until the angle of His was reached. We used two different staplers: (1) the Echelon Flex™ Powered Endopath® stapler (Ethicon Endosurgery, Inc., Somerville, NJ, USA) with gold cartridges, or (2) the Endo GIA™ (Covidien/Medtronic, Inc., Mansfield, MA, USA) with purple cartridges for the first 2 firings and blue cartridges for the remainder. The surgeon waited up to 20 s after closing the stapler before each firing process was initiated. Running suture was used to reinforcement staple line in some cases. If not – hemostatic clips were applied. Decision about the SLR was dependent on surgeon and was based on the intraoperative appearance of the staple line (visible bleeding etc.) and own experience.

The resected stomach specimen was then removed by enlarging one of the 13-mm ports. After testing for leaks with methylene blue dye (100 mL), a drain was placed alongside the staple line.

All patients after surgery were placed in post anesthesia care unit. Automatic 12 h blood pressure recordings were performed. In case of high blood pressure patients received single dose of 10 mg per orally Amlodipine. For analgesia we used morfine pomp (PCA), Paracetamol (Acetaminofen) and Metamizol. All patients underwent an additional test with methylene blue solution on postoperative day 1. If no leakage was detected, an oral diet was resumed. The patients were discharged on postoperative day 2.

### Bias

Observers were blind to the exposure and the data were collected retrospectively form medical records by single investigator. The risk of information bias is low. We analyzed large number cases of primary LSG (522) to minimize selection bias. According to the reports LSG constitute over 800 bariatric procedures per year in Poland [1, 12]. Thus, our data is representative. All patients underwent the same procedure according to standard surgical technique of LSG described above which eliminate referral bias. Study size was determined by the number of patients with HC who were matched with controls.

### Statistical Methods

Analysis was performed using SAS® software, University Edition (SAS Institute Inc., Cary, NC, USA). Continuous outcomes were analyzed using the paired **t** -test, or Wilcoxon signed ranks test. Dichotomous outcomes analyzed using McNemar’s test or Fisher’s exact test. The analysis of matched (dependent) data is different from unmatched (independent) data and is described in detail by Breslow and Day [13].

## Results

A total of 34 subjects (17 matched pairs) were included in the study. Table 1 presents the descriptive characteristics of the patients. The groups were closely matched as intended. In 15 cases the source of bleeding was identified. In 11 cases the bleeding was from staple line and in 4 cases from omentum. In 2 patients the location of bleeding was unknown – confirmed only by intraabdominal hematoma. Analysis of intra-operative findings with the method of staple line reinforcement revealed that bleeding was more common in patients after haemostatic clip application. Despite lack of statistical significance this finding was clinically important (Table 2). There was no difference in frequency of antihypertensive intervention between the groups.

**Table 1:** Demographic characteristics of patients

Characteristics (n)	Patients with HC (17)	Controls subjects (17)	*p*-value
Age (years)	44.3 ± 11.1	44.1 ± 10.8	0.260
BMI (kg/m2)	47.7 ± 6.4	47.1 ± 6.8	0.819
Gender			
Female	7 (41%)	7 (41%)	-
Male	10 (59%)	10 (59%)	
Comorbidities			
Hypertension	11 (65%)	12 (71%)	0.563
Diabetes	13 (76%)	12 (71%)	0.654
Dyslipidemia	2 (12%)	4 (23%)	0.414
Staple line reinforcement			
Haemostatic clips	12 (71%)	12 (71)%	-
Running suture	5 (29%)	5 (29%)	
Surgeon’s experience			
Low	8 (47%)	8 (47%)	-
High	9 (53%)	9 (53%)	
Source of bleeding			
Staple line	11	-	-
Omentum	2	-	
Unknown	4	-	
Antihypertensive intervention in postop unit?			
Yes	5 (31%)	4 (24%)	0.705
No	11 (69%)	13 (76%)

**Table 2:** Analysis of intra-operative findings with the method of staple line reinforcement

Source of bleeding	Staple line reinforcement		P - value
	Running suture	Haemostatic clips	
Staple line	2 (12%)	9 (53%)	0.413
Omentum	1 (6%)	1 (6%)	
Unknown	2 (12%)	2 (12%)	

**Fig. 1:**
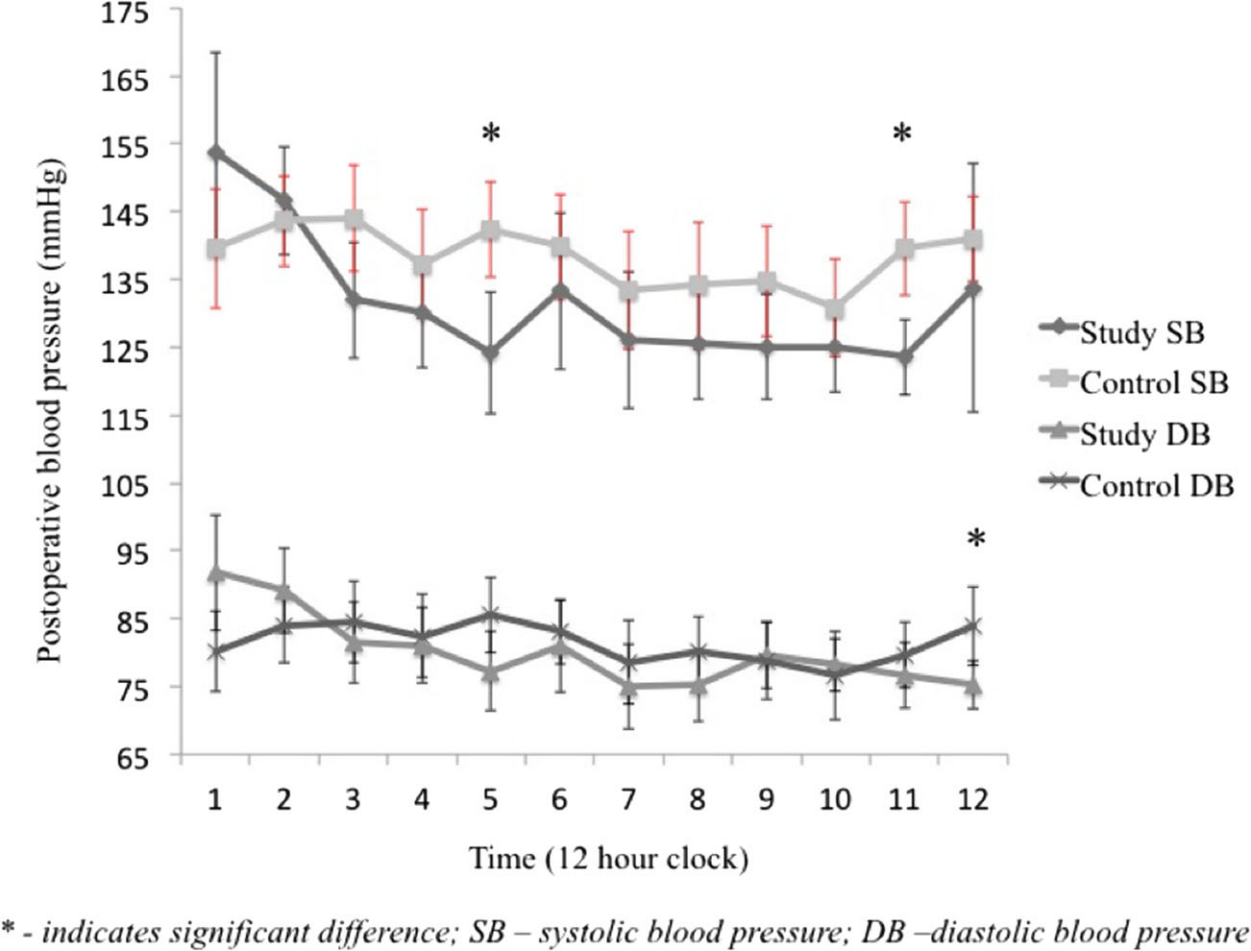
Mean 12 h systolic and diastolic blood pressure profiles for patients with HC (*n* = 17) and their matched controls (*n* = 17)

Patients who experienced hemorrhagic complications after LSG had non statistically significant decreased mean systolic blood pressure (mmHg) in 12 h observation (130.7 ± 12.9 versus 139.1 ± 10.8); *p* = 0.15; mean difference − 11.6 (95% CI -29.5 to 6.1). Mean 12 h diastolic pressure was also comparable (80.6 ± 7.2 versus 81.6 ± 7.2); *p* = 0.49; mean difference − 3.1 (95% CI -13.3 to 7.1). The detailed analysis of cases revealed significantly lower mean systolic blood (SB) pressure (mmHg) in the 5th hour of postoperative observation (126.2 ± 19.7 vs 142.4 ± 14.1; *p* = 0.03), mean difference − 16.9 (95% CI -31.8 to −1.96) and in the 11th hour of postoperative observation (123.1 ± 10.6 vs 139 ± 13.9; *p* < 0.01), mean difference − 17.4 (95% CI -29.0 to −5.8). Likewise, the mean diastolic blood pressure in the 12th hour of postoperative observation was significantly lower in cases (75.2 ± 6.9 vs 83.9 ± 11.5; *p* < 0.05;), mean difference − 12.9 (95% CI -25.6 to −0.1). Differences in the rest of blood pressure either SB or DB were not statistically significant. (Fig. 1)

## Discussion

This study reports 12-h blood pressure profiles of patients after Laparoscopic Sleeve Gastrectomy compared with closely matched control subjects drawn from the database of patients who underwent LSG between January 2013 and May 2015 in a single, high volume bariatric institution. The mean systolic and diastolic blood pressure measurements in 12-h observations were comparable. Controls subjects had higher systolic blood pressure in 5th and 11th postoperative hour and diastolic blood pressure in 12th postoperative hour. However the measurements in both groups were within normal limits and were not clinically significant. What is more, there was no difference in antihypertensive intervention between the groups.

Recently an observation was made that routine elevation of systolic blood pressure (SBP) to 140 mmHg at the end of stomach resection and oversewing the staple line minimized HC [14]. Elevation of SBP allowed better identification of intraoperative bleeding. The question about the blood pressure and a risk of bleeding in postoperative period should be raised. Our results did not show the association between hemorrhagic complications after LSG and high blood pressure levels in postoperative period. The findings are contrary to the popular belief that patients with hypertension in postoperative period are more likely to have bleeding complications. This belief was not supported by evidence [9, 15–17]. Our study is the first one, in which we have investigated this issue on bariatric patients. The take-home message derived from this study is that postoperative hypertension is not a risk factor for bleeding complications in patients after laparoscopic sleeve gastrectomy. Blood pressure levels within normal limits during postoperative observation are observed in patients who bleed after surgery.

The limitation of our study is the size of our study group. Bleeding complications in our study occurred in 4.03% of all cases and it matches the rates in other observations, ranging from 3% to 5% [7]. Since our database consists of 522 patients the amount of cases that qualified for the study should not be surprising.

## Conclusion

Compared with closely matched control subjects, patients with hemorrhagic complications after LSG have a decreased systolic blood pressure without statistical and clinical significance. Therefore the level of postoperative blood pressure is not a risk factor for hemorrhagic complications after LSG.
